# Fixed Flexion Contracture Can Successfully Be Addressed with Exact Preservation of the Femoral Joint Line and Only Minimal Increase of Tibia Resection in the Concept of Kinematically Aligned Total Knee Arthroplasty

**DOI:** 10.3390/jpm13050868

**Published:** 2023-05-21

**Authors:** Joaquin Moya-Angeler, Vicente J. León-Muñoz, Cristina Jimenez-Soto, Kim Huber, Bernhard Christen, Tilman Calliess

**Affiliations:** 1 Department of Orthopaedic Surgery, Hospital Universitario Reina Sofia, 30005 Murcia, Spain; jmoyaangeler@gmail.com (J.M.-A.);; 2 Instituto de Cirugía Avanzada de la Rodilla (ICAR), 30005 Murcia, Spain; 3 Medical School, University of Murcia, 30005 Murcia, Spain; 4 Articon Spezialpraxis für Gelenkchirurgie, 3013 Berne, Switzerland

**Keywords:** fixed flexion contracture, bone resection, kinematic alignment, robotic total knee arthroplasty, soft tissue release

## Abstract

The aims of this study were to evaluate the outcomes of patients undergoing kinematic alignment (KA) robot-assisted (RA) total knee arthroplasty (TKA) with and without preoperative fixed flexion contracture (FFC) and address whether additional resection of the proximal tibia is required to address FFC. A retrospective review from 147 consecutive patients who received an RA-TKA with KA and a minimum one-year follow-up was performed. Preop and postop clinical and surgical data were collected. Groups were set based on preoperative extension deficits: group 1 (0–4°) (n = 64), group 2 (5–10°) (n = 64) and group 3 (>11°) (n = 27). There were no differences in patient demographics among the three groups. In group 3, the mean tibia resection was 0.85 mm thicker than group 1 (*p* < 0.05) and the preoperative extension deficit was improved from −17.22° (SD 3.49) preop to −2.41° (SD 4.47) postop (*p* < 0.05). Our results demonstrate that FFC can successfully be addressed in the RA-TKA with KA and rKA and that no additional femoral bone resection is needed to achieve full extension in patients with preoperative FFC when compared with patients without FFC. Only a slight increase in the amount of tibial resection was observed, but this was less than one millimetre.

## 1. Introduction

Fixed flexion contracture (FFC) is a common problem that is frequently associated with end-stage knee osteoarthritis (OA) [1]. In unicondylar knee arthroplasty it is often referred as a relative contraindication, as it cannot be addressed properly with limited ability for soft tissue releases and alterations to the joint line. However, in total knee arthroplasty (TKA) there is also an increased risk for a remaining FFC, causing poor function and low patient satisfaction [2,3,4].

Since the 1970s, mechanical alignment (MA) has been established as the standard of care in TKA, correcting bone deformities and contractures to neutral [5]. Therewith, adaptions and releases of the soft tissue envelope are necessary to achieve a balanced and stable knee. The target for mechanical alignment is a straight overall leg axis, a rectangular and symmetric flexion and extension gap and a full extension. One of the most common methods described to address an FFC in mechanically aligned TKA is to perform more distal femur resection combined with soft tissue releases at the posterior capsule and posterior cruciate ligament and posterior osteophyte removal [6,7,8]. The additional resection of distal femoral bone, however, has several downsides, including a longer surgical time when re-recutting is required, increased risk of bleeding, infection [9] and deep vein thrombosis (DVT) [10] and an altered higher joint line (JL). This JL proximalisation could ultimately lead to a TKA with mid-flexion instability [11,12], which is one of the major causes of early revision after TKA [13]. Additionally, the patella position is altered and thus the lever arm of the quadriceps muscle is decreased [11,12].

Over the past decades, several alternative alignment philosophies were introduced for TKA [14,15,16,17]. Most of them aiming for a more anatomic and individualized positioning of the components to the patient’s pre-arthritic knee. The common ground is to reduce soft tissue releases and to minimise alterations in the knee kinematics with a more physiological loading and ligament stability. One of the best-defined concepts in this context is the kinematic alignment (KA) philosophy or restricted KA (rKA) that has boundaries with respect to the overall leg axis and joint line obliquity [5]. The primary aim of KA/rKA is to restore the pre-arthritic surface anatomy of the knee as closely as possible as well as the kinematic axes of the native knee with the prosthesis. Therewith, also the natural ligament tension and joint stability is restored. These primary flexion and extension axes, upon which the alignment philosophy is based, are located in the distal femur condyles and are defined by their surface anatomy. Thus, any additional distal femoral resection or joint line shift that is similar to classical mechanically aligned TKA counteracts the idea of a kinematic alignment [14]. This bone-sparing resection approach is particularly relevant in the case of preoperative FFC, and one research question is whether or not FFC can be successfully addressed without additional distal femoral bone resection and or classic soft tissue releases. There is only very limited data evaluating this topic. Recent data suggest that KA, in general, is bone-sparing compared with MA, allowing for joint line preservation and lesser soft tissue damage in different osteoarthritic deformities [16]. However, minimal data are available on the effectiveness of addressing FFC without additional femoral bone resection and the possible need for additional tibia resection in the concept of KA and rKA TKA.

Especially with the use of modern technologies such as image-based or image-less robotics, the idea of alternative alignment methods has gained more interest [18]. The individual alignment target can be determined and planned based on the structural information of the bone model as well as on the soft tissue information and knee kinematics that are recorded during surgery. That makes the procedure more precise, accurate and able to achieve the set alignment target and more reproducible and understandable, as all parameters are displayed on the system [18]. Thus, we routinely use robotic assistance to achieve KA or rKA for TKA, with precise depiction of the resection levels to achieve a true measured resection, especially on the femur to restore the femoral surface and co-align the prothesis to the kinematic axes. The tibia is then co-aligned to the femur based on the soft tissue balance in extension. Tibia resection height is determined by the extension gap to achieve full extension [15].

Consequently, we performed a retrospective study to evaluate the outcomes of patients undergoing rKA robot-assisted (RA) TKA with and without preoperative FFC and whether or not additional resection of the proximal tibia is required to address FFC. We hypothesized that FFC could successfully be addressed without altering the femoral joint line but may require more tibia resection.

## 2. Materials and Methods

A retrospective review of prospectively collected data from 147 consecutive patients who received an RA-TKA with KA or rKA at our institution was performed. The minimum follow-up for clinical data was one year post surgery. Informed consent was obtained for data analysis from all subjects involved in the study.

Two senior surgeons performed all surgeries in the period from October 2018 to October 2021. The MAKO image-based robotic system was used for all cases, as was the STRYER TRIATHLON posterior-stabilized (PS) knee prosthesis. All cases were pre-planned with the proprietary software by the surgeon based on the preoperative segmented CT data. First, the femoral component was adjusted following the KA principle, setting the distal and posterior resection level to 6 mm. This is to restore the native joint line with the 8 mm-thick prosthesis (6 mm bone resection based on CT scan plus 2 mm cartilage thickness = 8 mm total resection volume). The boundaries for kinematic alignment on the femur were a maximum of 3° valgus angle and a resulting relative internal rotation of the femur with respect to the native trochlea orientation. In these cases, adjustments were made to a restricted kinematic alignment philosophy as follows: the medial distal and posterior femur resection were always left at 6 mm, and only the lateral ones were adapted (less resection) so that the medial column was always reconstructed anatomically following the KA principle. On the tibia, only a conservative precut was planned at a 3.5-to-4.5 mm resection level with a conservative tibia vara between 0 and 2° and a conservative tibia slope ranging from 0 to 3° best matching the native anatomy. During surgery, the positioning of the arrays, surgical approach and bone registration was according to the in-house surgical standard and the standard MAKO procedure. First in surgery, after bone registration, the preoperative extension deficit was recorded by putting the knee in full extension without pressure. Then, the correct femoral resection level was verified by mapping the remaining cartilage level in areas without osteoarthritic wear. Minor adjustments in the preplanning were made based on this. After that, all femoral bone cuts plus the tibia precut were conducted with the assistance of the haptic robotic arm. Then, all osteophytes were approached and meticulously removed with the help of image-based navigation. As in the concept of KA, no classic soft tissue releases were performed, but resection of the posterior cruciate ligament for the PS prosthesis and a detachment of the posterior capsule on the femur in case of FFC were. After these steps, the flexion and extension gaps were recorded with the help of the STRYKER MONOGRAM BALANCER and MAKO ligament balancing software tool. The target zone for the gap balance was a symmetric extension gap of 18–20 mm height (±1 mm difference medial to lateral accepted). In cases of medial tightness, tibia orientation was put in more varus and recut with a maximum of 5° tibia vara. In cases of remaining tightness medial, the tibia plateau was downsized by one size, aligned to the very lateral boarder of the plateau and the medial overhanging bone was resected to indirectly release the medial joint space (only one posttraumatic case in this series). In cases of lateral tightness, the tibia was adjusted to be more valgus, to a maximum of 1° valgus for the overall limb alignment (thus, depending on the femur orientation). In cases with remaining lateral tightness, minor releases on the iliotibial band and lateral capsule were performed to achieve a symmetric extension gap. In cases of symmetric tightness of the extension gap, the tibia resection level was increased to meet the target zone. The flexion gap target was an isometric balance medially to the extension gap ±2 mm, whereas laterally, the natural laxity up to a 5 mm difference from medial was accepted. After these adaptions, the virtually planned adjusted tibia position was recut with robotic assistance. This situation was then evaluated with a trail prosthesis to ensure full (0°) extension. In cases of a remaining extension deficit, the tibia was recut stepwise in 1 mm steps until full extension could be reached. When satisfactory knee balance and range of movement was achieved, the prosthesis was positioned accordingly.

For data analysis, the definite bone resection levels on the medial and lateral distal and posterior femur and proximal tibia were recorded at the end of surgery. For statistical analysis, the maximum resection levels were used and the summation of the maximum resection of the distal and posterior femur and tibia was calculated.

Additional to the surgical data, baseline patient demographics (age, sex, side, ASA score and BMI) were recorded and analysed using descriptive statistics. The primary outcome parameter of the study was the pre- and postoperative active range of motion (ROM) at one-year follow-up. Additionally, the preoperative clinical situation was evaluated with the standardized Knee Society score (KSS), Oxford knee score (OKS), knee injury and osteoarthritis outcome score (KOOS) and the EQ5d questionnaire. At one year post-surgery, there was a clinical examination for the active ROM and again the collection of all mentioned scores plus the forgotten joint score (FJS12).

For data analysis, patients were divided into three groups based on their preoperative extension deficit: group 1 (0–4°) (n = 64), group 2 (5–10°) (n = 64) and group 3 (>10°) (n = 27).

We used the Statistical Package for the Social Sciences (SPSS), version 25 for Windows (SPSS, Inc., Chicago, IL, USA) to perform the statistical analysis. To assess the differences between the variables of the three groups established based on the degree of preoperative extension deficit, we used one-way analysis of variance (ANOVA) and Pearson’s chi-square test to compare proportions. To determine the significance of the difference in the different variables after the intervention, we performed paired *t*-tests. We used the Shapiro–Wilk test to check that the *p* values were above the significance level of 0.05, with the null hypothesis that the data fitted a normal distribution being accepted.

## 3. Results

The descriptive characteristics of the variables of the three groups are shown in Table 1. There was no significant difference between the three groups regarding ASA score, age or BMI. Only male patients were overrepresented in group 3 (those with FFC of more than 10°).

As described in the methodology, the femoral resection level was pre-set following the principle of KA to a symmetric 6 mm bone resection. It was only adjusted to match the cartilage surface or to meet the natural trochlear orientation (rKA). Thus, femoral resection showed no significant difference between all three groups, as displayed in Table 2 (*p* > 0.05). Lateral femur resection was on average less then medial in all groups due to the boundaries for KA and the resulting adjustments to rKA as described.

**Figure 1:**
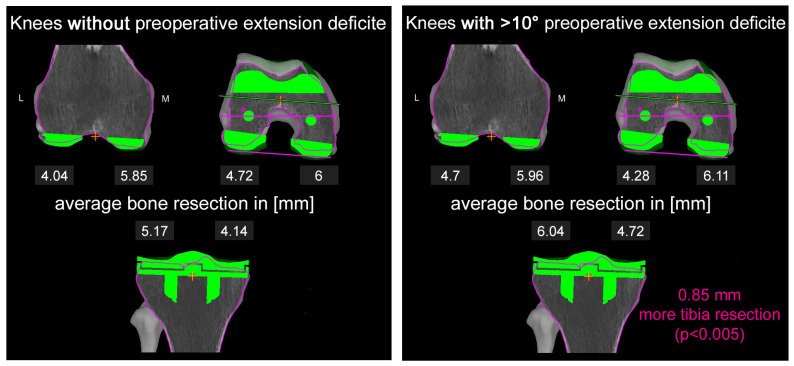
Graphical display of the average bone resection levels on (**left**) to (**right**) distal lateral and medial femur, posterior lateral and medial femur and proximal lateral and medial tibia for group 1 (**left**) and group 3 (**right**). Pink represents the mean difference in tibial (Tibial max.) resection among knees without preoperative extension deficit (group 1) and knees with over 10° of preoperative extension deficit (group 3). (L) lateral, (M) medial.

The only significant difference was found for the tibia resection height. In group 3, those with FFC of more than 10°, the mean tibia resection was 0.85 mm more than for group 1 (*p* < 0.05) (Figure 1).

By definition, the preoperative active extension differed between all three groups. Additionally, active flexion and, consequently, the ROM was significantly lower in group 3 compared with groups 1 and 2 (*p* < 0.001). This difference also remained postoperatively with, on average, 10° less active ROM for group 3 (*p* < 0.001). Moreover, there was still an increased risk for FFC postoperatively in group 3 (*p* < 0.001); however, the preoperative extension deficit could be reduced from −17.22° (SD 3.49) preop to −2.41° (SD 4.47) postop (*p* < 0.05). No differences were observed in all other postoperative outcome parameters, as shown in Table 3.

## 4. Discussion

This study evaluated the amount of bone resection performed to address FFC during RA-TKA with a KA and rKA philosophy in patients presenting with different degrees of preoperative FFC. Our results demonstrated that FFC could successfully be addressed in the RA-TKA with KA and rKA and that no additional femoral bone resection nor joint line elevation was needed to achieve full extension in patients with preoperative FFC compared with patients without FFC. Only a slight increase in the amount of tibial resection was observed, but this was less than one millimetre. Clinical outcomes showed no differences between the analysed subgroups at one-year follow-up.

The adequate management of FFC is a crucial factor in achieving satisfactory results after TKA [2,3,19]. In the MA-aligned TKA, this has been traditionally managed with a combination of soft tissue releases and additional distal femoral bone resection [8,20,21]. Several studies have reported the consequences of these gestures, which include a longer operative time, a higher risk of infection and DVT and the rise of the JL, which ultimately can lead to increased coronal laxity in mid-flexion [9,10,19,22,23].

KA has emerged as an alternative alignment technique to MA with equivalent or slightly better results [14,16]. Since the aim of KA is to restore the physiological native anatomy of the knee joint, it allows a more conservative approach during TKA with less bone resection and soft tissue release. As all our cases were performed with KA or rKA alignment, as described in the methodology, the femoral component was first adjusted to restore the native joint line (at least on the medial side), thus we did not perform any additional resection in the femur to address FFC. The additional bone resection to correct FFC was performed in the tibia and was only 0.85 mm, which is not significant compared with the additional femoral bone resection required to correct FFC in the MA-aligned TKA (from 5 to 9 mm) [24,25,26]. Our results also showed that some flexion contracture could remain postoperatively, especially in cases of severe preoperative FFC. However, this was significantly less than the pre-existing FFC and has also been described to happen when the traditional method to address FFC is applied (performing more distal femur resection combined with soft tissue releases and osteophyte removal with an elevation of the joint line) [6,7,8]. An et al. [16] recently compared the ability of MA and KA to correct coronal malignment and FFC. They concluded that using a KA philosophy in TKA results in achieving extension range of motion and soft tissue balance goals with less bone resection and fewer soft tissue releases. Although our study did not account for an MA group for comparison, it also demonstrated that no additional bone resection was required in the femur to achieve full extension in patients undergoing KA or rKA RA-TKA with preoperative FFC when compared with those with no FFC. This, in turn, results in greater preservation of bone stock and a lower risk of altering the joint line, which optimizes the range of motion, decreasing component wear and postoperative pain. Although we did not specifically measure the joint level change or laxity before and after TKA, several studies have demonstrated that the additional resection of the distal femur can raise the joint line and cause mid-flexion coronal laxity after TKA [12,27,28]. Additionally, since no soft tissue releases were required, this may have also contributed to a shorter operative time, which was not evaluated in this study but has been previously demonstrated by others [15]. Other benefits of not performing soft tissue releases are the maintenance of the proprioceptive function and balance [29], which contribute to better function, faster patient rehabilitation and higher patient satisfaction.

Our data show that in cases of severe FFC, additional tibia resection could be necessary to achieve full extension during surgery. However, as the extension deficit is usually an extension gap problem only, this approach could potentially lead to a flexion extension gap mismatch and a relative flexion gap laxity. This is why the classic MA approach addresses the distal femur with additional resection volume to only address the extension gap. This flexion and extension mismatch was not evaluated in detail in our study; however, we did not find any clinical differences between the subgroups in any outcome parameter at one-year follow-up. The OKS is in the “excellent” range for both groups 1 and 3; additionally, the FJS is above average compared with the current literature on MA TKA. Furthermore, the additional resection on the tibia was only about 0.83 mm with a standard deviation of 1.2 mm. We interpreted this as clinically not relevant, at least in the context of PS prosthesis with a stabilizing mechanism for the flexion gap and the femoral rollback.

This study has a few limitations. First, the absence of a control group in which an MA-TKA was performed to address FFC. Second, we did not directly measure changes in joint line level preop and postoperatively but only the resected bone volumes. This could potentially be influenced by the position of the landmarks or massive bone wear. However, the protocol to position the landmarks was highly standardized and controlled by two independent observers and bone wear is less likely on the femur than on the tibia in end stage (varus) osteoarthritis. No detailed information on the resulting gaps is included in this study. Third, this was a retrospective study of prospectively collected data in a consecutive case series. Higher-quality research studies should validate the results. In addition, we only used one implant design (PS implant where better posterior releases can be performed) for this study. Other implant designs could yield different results.

## 5. Conclusions

A full extension can be achieved without resecting additional distal femur in preoperative FFC in kinematically aligned TKA. On average, the tibia resection level may need to be adjusted but with less than 1 mm additional bone resection.

## Figures and Tables

**Table 1:** Baseline characteristics of the study groups. Patients were divided into three groups based on their preoperative extension deficit: group 1 (0° to 4°), group 2 (5° to 10°) and group 3 (>10°).

		Group 1 (n = 64)	Group 2 (n = 56)	Group 3 (n = 27)	*p*-Value
Gender ^b^	Female cases	40 (62.5%)	36 (64.3%)	10 (37%)	0.042
	Male cases	24 (37.5)	20 (35.7%)	17 (63%)	
ASA ^b^	ASA 1	7 (10.9%)	7 (12.5%)	6 (22.2%)	0.316
	ASA 2	46 (71.9%)	33 (58.9%)	15 (55.6%)	
	ASA 3	11 (17.2%)	16 (28.6%)	6 (22.2%)	
Side ^b^	Left	39 (60.9%)	33 (58.9%)	9 (33.3%)	0.041
	Right	25 (39.1%)	23 (41.1%)	18 (66.7%)	
Age ^a^		66.98 (8.54)	68.89 (7.39)	66.56 (7.83)	0.318
BMI (kg/m^2^) ^a^		27.35 (4.51)	28.73 (5.94)	28.78 (4.42)	0.263

Baseline characteristics of the study groups: ASA, American Society of Anesthesiologists physical status classification (ASA I: normal healthy patient; ASA II: patient with mild systemic disease and ASA III: patient with severe systemic disease); BMI: body mass index; values are shown as ^a^ mean and (standard deviation) or ^b^ n and (%). All differences were considered significant at a probability level of 95 % (*p* < 0.05).

**Table 2:** The study groups’ femoral and tibial intraoperative resection levels (in mm). Patients were divided into three groups based on their preoperative extension deficit: group 1 (0° to 4°), group 2 (5° to 10°) and group 3 (>10°). Highlights denotes differences in the femoral distal (0.08) and tibial maximum resection levels (0.85) among knees without preoperative extension deficit (group 1) and knees with over 10° of preoperative extension deficit (group 3).

	Group 1 (n = 64)	Group 2 (n = 56)	Group 3 (n = 27)	*p*-Value
Femoral distal medial	5.85 (0.44)	5.87 (0.73)	5.96 (0.47)	0.757
Femoral distal lateral	4.04 (1.68)	4.32 (1.47)	4.7 (1.48)	0.262
Femoral distal max.	5.94 (0.36)	5.95 (0.59)	6.02 (0.38)	0.755
Femoral posterior medial	6 (0.38)	6.09 (0.5)	6.11 (0.34)	0.478
Femoral posterior lateral	4.72 (1.2)	4.21 (1.23)	4.28 (1.51)	0.153
Femoral posterior max.	6 (0.38)	6.09 (0.5)	6.11 (0.34)	0.478
Tibial medial	4.14 (1.28)	4.14 (1.62)	4.72 (1.62)	0.261
Tibial lateral	5.17 (0.82)	5.09 (1.32)	6.04 (1.44)	0.005
Tibial max.	5.39 (0.73)	5.46 (0.78)	6.24 (1.2)	0.001

**Table 3:** Mobility range and outcomes on the different scores preoperatively and at one-year follow-up. Patients were divided into three groups based on their preoperative extension deficit: group 1 (0° to 4°), group 2 (5° to 10°) and group 3 (>10°). Highlights denotes differences that were statistically significant.

	Group 1 (n = 64)	Group 2 (n = 56)	Group 3 (n = 27)	*p*-Value
Preoperative active extension (°)	0 (0)	−7.41 (2.52)	−17.22 (3.49)	<0.001
Preoperative active flexion (°)	126.25 (9.64)	119.38 (12.03)	109.63 (16.58)	<0.001
Preoperative active ROM (°)	126.25 (9.64)	111.96 (12.78)	92.41 (16.89)	<0.001
Preoperative KSS	109.25 (21.4)	100.75 (16.97)	96 (25.43)	0.01
Preoperative mean KOOS	42.35 (16.25)	39.25 (12.38)	39.59 (13.67)	0.563
Preoperative Oxford knee Score	24.81 (7.9)	24.71 (6.54)	26.96 (8.49)	0.392
Preoperative EQ5	0.59 (0.29)	0.65 (0.25)	0.65 (0.28)	0.48
Postoperative active extension (°)	−0.31 (1.22)	0 (0)	−2.41 (4.47)	<0.001
Postoperative active flexion (°)	129.69 (8.06)	129.29 (9.26)	122.04 (11.12)	0.001
Postoperative active ROM (°)	129.38 (8.33)	129.29 (9.26)	119.63 (12.47)	<0.001
Postoperative KSS	186.08 (18.72)	186.45 (17.04)	185.26 (13.57)	0.957
Postoperative mean KOOS	78.72 (16.76)	80.39 (14.7)	78.1 (19.02)	0.798
Postoperative Oxford knee Score	41.31 (7.31)	39.91 (7.76)	41.4 (7.05)	0.541
Postoperative EQ5	0.89 (0.15)	0.89 (0.16)	0.83 (0.24)	0.333
FJ12 score	66.99 (27.81)	69.24 (25.9)	68 (29.3)	0.909

Mobility range and outcomes: ROM, range of motion; KSS, Knee Society score (zero points = the worst score and 100 points = the best score); KOOS, knee injury and osteoarthritis outcome score (KOOS assesses five subscales: pain, other symptoms, activities of daily living, function and sports/recreational activities and quality of life). The Oxford knee score results were presented in a continuous score ranging from zero (most severe symptoms) to 48 (least symptoms). EQ5 describes a self-perceived health status and health-related quality of life ranging from zero (death) to 1 (perfect health). FJ12, forgotten joint score 12 for the knee questionnaire, measurement of patient-reported outcomes quantifying the patient’s ability to forget the artificial joint in everyday life (zero points = worst score and 100 points = the best score). Values are shown as mean and (standard deviation). All differences were considered significant at a probability level of 95 % (*p* < 0.05).

## Data Availability

Not applicable.
